# Media Health Literacy in Spanish Nursing Students: A Multicenter Cross-Sectional Study

**DOI:** 10.3390/nursrep14030189

**Published:** 2024-09-22

**Authors:** Noelia Navas-Echazarreta, Pedro José Satústegui-Dordá, Francisco José Rodríguez-Velasco, María Eva García-Perea, Antonio Martínez-Sabater, Elena Chover-Sierra, María Luisa Ballestar-Tarín, Pablo Del Pozo-Herce, Silvia González-Fernández, Regina Ruiz de Viñaspre-Hernández, Michal Czapla, Raúl Juárez-Vela

**Affiliations:** 1 Doctoral Program in Health Sciences and Sports, University of Zaragoza, 50009 Zaragoza, Spain; noelia.navas@unirioja.es; 2 Predoctoral Researcher, University of La Rioja, 26006 Logroño, Spain; 3 SAPIENF (B53_23R) Research Group, Department of Physiatry and Nursing, Faculty of Health Sciences, University of Zaragoza, c/ Domingo Miral s/n, 50009 Zaragoza, Spain; 4 Biopsychosocial Research Group, Faculty of Medicine and Health Sciences, University of Extremadura, 06006 Badajoz, Spain; fcorodriguezv@unex.es; 5 Head of Department in Nursing, Faculty of Medicine, Madrid Autonomus University, 28049 Madrid, Spain; eva.garcia@uam.es; 6 Nursing Care and Education Research Group (GRICE) GIUV2019-456 Nursing Department, Faculty of Nursing and Podology, 46010 Valencia, Spain; elena.chover@uv.es (E.C.-S.); m.luisa.ballestar@uv.es (M.L.B.-T.); 7 Care Research Group (INCLIVA) Clinic Hospital of Valencia, 46010 Valencia, Spain; 8 Department of Psychiatry, Fundación Jimenez Diaz University Hospital, 28040 Madrid, Spain; pablo.pozo@quironsalud.es; 9 Faculty of Medicine, University of Salamanca, 37007 Salamanca, Spain; sigofe@usal.es; 10 GRUPAC Research Group, Department of Nursing, University of La Rioja, 26006 Logroño, Spain; reruizde@unirioja.es (R.R.d.V.-H.); michal.czapla@umw.edu.pl (M.C.); raul.juarez@unirioja.es (R.J.-V.); 11 Department of Emergency Medical Service, Wrocław Medical University, 51-616 Wrocław, Poland

**Keywords:** media health literacy, nursing education, socio-economic factors, socio-educational factors, Spain

## Abstract

Background: Amidst current misinformation, media literacy is an essential competency for nursing professionals. This study aimed to analyze the level of media health literacy among Spanish undergraduate nursing students, stratifying the results by gender, region, and other associated factors. Methods: A cross-sectional study was conducted at five Spanish universities (N = 416) using the Spanish version of the Media Health Literacy (MeHLit-SV) Questionnaire. Results: Students presented an average media health literacy score of 48.73 points. The media health literacy level was significantly higher among students from universities in the central and northeastern regions of Spain (ANOVA Test, *p* = 0.0002), those who had previously studied in a city (ANOVA Test, *p* = 0.001), those who combined their studies with employment (ANOVA Test, *p* = 0.001), and those residing in communities with fewer than 500 inhabitants (ANOVA Test, *p* = 0.001). No differences were found based on gender. Conclusions: The media health literacy level of the students was deficient and varied according to socio-economic and socio-educational factors. Understanding the literacy level of future nurses and promoting the inclusion of this competency in their education will enable them to become leaders in improving the population’s health self-care.

## 1. Introduction

Currently, the concern regarding misinformation is growing and has continued to increase following the COVID-19 pandemic. This upward trend was already evident in the years preceding the arrival of the virus, during which the impact of misinformation on health and its underestimation throughout history progressively gained recognition [1,2]. Furthermore, it is important to note how the vast amount of health information available across various media platforms creates a conducive environment for the publication of erroneous or misleading content, thereby generating a context where complete verification is materially impossible [3].

Misinformation is a multidisciplinary issue that goes beyond the field of communication. Health is a major topic of interest for the public, as seen in the high volume of publications in various media. Healthcare professionals, academics, and scientists agree that misinformation poses a threat to both individual and public health, calling for immediate action [2,4]. As a result, public health is increasingly affected, making it essential to understand and address this issue [5].

In the current context, media, particularly digital platforms, serve as the primary source of information for the public. Among the most sought-after topics is health-related information, which generates substantial interest. Thus, it is essential that citizens develop specific competencies, such as media health literacy, to critically evaluate and understand the information they consume [6]. Media health literacy encompasses the skills or abilities that individuals acquire to understand, analyze, and appropriately use health information disseminated through various media channels. In contrast to health literacy, which focuses on acquiring the ability to access, comprehend, and use health information effectively [7], media health literacy emphasizes the critical analysis of media messages related to health, which the public is exposed to daily and may contain misinformation or inaccuracies that could negatively affect their health [8].

Given the impossibility of ensuring that all information consumed by the public is truthful and accurate, media health literacy emerges as an effective response to this misinformation ecosystem [9,10]. The ability to critically analyze information is crucial for making informed decisions about individual and community health. A higher level of media health literacy is associated with positive health outcomes, as it enables people to adopt healthier lifestyles, access more appropriate healthcare, and actively participate in managing their well-being. Therefore, improving media health literacy is essential for promoting population health [8].

In this regard, public concern about misinformation is growing, highlighting the need to develop prevention strategies. According to reports from the Eurobarometer [11], Spain ranks as the second country in the European Union with the highest concern about misinformation (82%), surpassed only by Malta. In the rest of Europe, this percentage is lower (around 70%) and is partly related to the academic and educational responses that have been implemented over the years. According to the Ministry of Foreign Affairs, the European Union, and Cooperation [12], academic institutions are key actors in the fight against misinformation, along with strategic and digital communication. Additionally, the North Atlantic Treaty Organization (NATO, 1949) is also active in this conflict, which adversely affects public health, implementing programs and initiatives that promote media literacy and the analysis of information disseminated in the digital sphere, similar to the efforts of the European Union [11,12,13,14].

To understand the importance of media health literacy among nursing students in Spain, it is crucial to consider both the cultural and political context of the media as well as the university model in which nursing education is developed. On one hand, the Spanish media system follows the polarized pluralist or Mediterranean model proposed by Hallin and Mancini [15]. This model, characterized by weak journalistic professionalism, high political parallelism, and strong state intervention, has resulted in a media landscape marked by low circulation of printed newspapers and a dominant presence of television, the internet, social media, and, to a lesser extent, digital press in everyday life [16]. Overall, Spanish media represents a key source of health-related information for most of the population. However, the health information disseminated through these media channels frequently contains numerous informational inaccuracies, negatively affecting the health of a population that largely lacks proper media literacy [17,18,19].

This described media environment influences public perceptions and is largely responsible for the population’s misunderstanding of health and self-care. Nursing students are immersed in this context. Regarding nursing education in Spain, it combines theoretical classroom attendance with clinical placements from early stages [20]. For this reason, incorporating media health literacy into the nursing curriculum would not only improve clinical practice from undergraduate levels but also positively impact self-care and the care of those around them [6]. Based on this, it would be advisable to integrate all proposals aimed at ensuring that nursing students achieve high levels of media health literacy into the nursing degree curriculum.

Thus, empowering the public to identify the presence of informational disorders through proper analysis of the information that constitutes their media diet represents an achievement at the health, academic, and social levels. Healthcare professionals are a group particularly affected by the overwhelming amount of health-related misinformation, which negatively impacts the self-care of their patients [5,21,22].

Nurses play a crucial role in improving the media health literacy of the population due to the activities they can engage in during their daily practice. Nurses interact directly and continuously with patients, providing opportunities to educate, inform, and empower individuals to understand and effectively use health information [23,24,25]. Therefore, it is essential for nurses to be media literate and to develop their symbolic capital [26], being recognized by the public as trustworthy individuals when analyzing and communicating health information [5,21,22].

In this context, the underlying question guiding the present research is to assess the level of media health literacy among nursing students as future healthcare professionals. Thus, the objective of this study was to analyze the level of media health literacy among students in the Bachelor of Nursing program at various Spanish universities, stratifying the results by gender and region, and identifying other factors that may be associated.

## 2. Materials and Methods

### 2.1. Study Design

During the months of March and April 2024, we conducted an observational, descriptive cross-sectional study. Our study adhered to the STROBE checklist guidelines tailored for cross-sectional studies [27].

### 2.2. Population and Scope of This Study

A multicenter study was conducted at five Spanish universities with second-year nursing students who completed the questionnaire fully and correctly. Specifically, the sample consisted of students from the University of La Rioja (n = 85)—located in the northern region of Spain—the University of Zaragoza (n = 118)—in the northeastern part of the country—the University of Valencia (n = 113)—in the Mediterranean region—the University of Extremadura (n = 70)—in the southern region—and the Autonomous University of Madrid (n = 30)—in the central region of the Iberian Peninsula. The results were obtained from the questionnaire administered during the months of March and April 2024. From the total number of students in the study population (N = 772), the sample calculation for a 95% confidence level and a 5% margin of error yielded a sample size of 258 students. The final sample consisted of 416 participants.

The inclusion criteria pertained to second-year students from the University of La Rioja, the University of Zaragoza, the University of Valencia, the University of Extremadura, and the Autonomous University of Madrid who provided informed consent. The exclusion criteria included participants with incomplete or incorrectly completed questionnaires, which were therefore not useful for this study.

### 2.3. Data Collection Instrument and Procedure

The information was collected in person using an online form on the Google Apps for Education (GAFE) platform, which was shared via a QR code projected in the classroom. This software is a suite of tools and services from Google designed for educational institutions, enabling safe collaboration and learning. This research study was conducted with the approval of the Ethics Committee of the University of La Rioja (Spain) under verification number: CE 69/2024. The data were protected, and each student could access the questionnaire only once. Upon accessing the questionnaire, participants were asked to sign their consent to participate in this study and were provided with a brief explanation of the guidelines for proceeding and accessing the questionnaire. The questionnaires were recorded and coded using random numbers and letters.

Media health literacy was assessed using the Spanish version of the Media Health Literacy scale (MeHLit-SV) [28]. With a Cronbach’s alpha of 0.936 for the total score, this scale consists of 21 items with 5 response options (0 = never, 1 = rarely, 2 = sometimes, 3 = most of the time, and 4 = always). Scores range from 0 to 84, with higher scores indicating higher levels of media health literacy. The Spanish version of the Media Health Literacy Questionnaire (MeHLit-SV) [28] confirms the 21-item structure of the original model by Nazarnia et al. [29], with a Cronbach’s alpha of 0.91.

### 2.4. Study Variables

The primary variable of this study was the level of media health literacy among nursing students, measured using the Spanish version of the Media Health Literacy scale (MeHLit-SV) [28]. Secondary or explanatory variables included aspects related to the socio-demographic characteristics of the students (gender, age, population center, environment, and type of family), their academic education (type of institution and choice of degree), their economic level (employment status and financial situation), the technological devices they used to obtain information, as well as their perceived level of happiness and health.

### 2.5. Statistical Procedures

The analysis included descriptive statistics to summarize quantitative values, encompassing measures such as the mean, standard deviation, median, coefficient of variation, and asymmetry. Categorical variables were depicted through both absolute and relative frequencies.

Normality of distributions was assessed via the Kolmogorov–Smirnov test, with a significance threshold set at 0.05, corresponding to a 95% confidence interval. Values below this threshold (*p* < 0.05) were deemed statistically significant. Univariate and bivariate analyses were conducted, with the latter exploring relationships between quantitative and qualitative variables. Mean differences between continuous variables (such as age and media health literacy) were examined.

Prior to conducting tests involving multiple groups, normality and equality of variances were assessed. For equal variances, an ANOVA test was utilized.

The statistical analysis was carried out using the RCommander program (version 3.6.1).

## 3. Results

### 3.1. Socio-Demographic Characteristics

Table 1 presents the socio-demographic characteristics of the study population. The students had a mean age of 21.6 years and were enrolled in the Bachelor of Nursing program at the universities of La Rioja (20.43%), Zaragoza (28.37%), Valencia (27.16%), Extremadura (16.83%), and the Autonomous University of Madrid (7.21%). The sample composition was predominantly female (83.89%), with a majority of women educated in an urban environment (74.52%), primarily from population centers with more than 5000 inhabitants (86.3%).

Additionally, 83.89% of the students came from a biparental nuclear family, and 64.66% had a sibling. The majority of the students (62.02%) had completed their primary and secondary education in public educational institutions, and nearly the same percentage (63.94%) reported having more money than necessary to make ends meet. Although 61.78% of the students balanced their studies with paid employment, only 6.25% reported experiencing financial difficulties. Regarding self-perceived health, a substantial majority of the students (89.19%) stated that they were in good or very good health.

### 3.2. Level of Media Health Literacy

**Figure 1:**
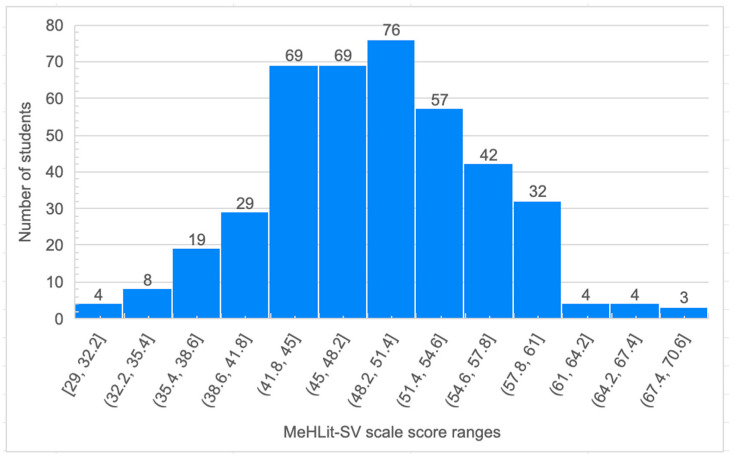
Distribution of media health literacy score (MeHLit-SV) in students.

The students demonstrated a mean score of 48.73 points on the MeHLit-SV scale for media health literacy. The distribution of the students’ scores did not follow a normal distribution (Kolmogorov–Smirnov test, *p* = 0.026), although it visually appeared to be symmetrical (Figure 1). Among the students, 51.44% (n = 214) scored between 41 and 51 points on the MeHLit-SV scale. Additionally, 14.42% (n = 60) scored below 42, with a minimum score of 29 points, while 34.13% (n = 142) exceeded 51 points, achieving a maximum score of 69 (Table 2).

As shown in Table 2, statistically significant differences were found in media health literacy scores based on the university attended (ANOVA, *p*-value = 0.0002). Specifically, students from the Autonomous University of Madrid demonstrated the highest literacy, with a mean score of 50.33 points, while students from the University of Extremadura obtained the lowest score (45.60 points). However, the level of media health literacy did not differ by gender (ANOVA, *p* = 0.713).

### 3.3. Relationship between Level of Media Health Literacy and the Socio-Demographic Variables

Table 3 presents the results of the analysis of the Media Health Literacy variable on the MeHLit-SV scale, stratifying the results according to the demographic variables of interest for this study. In this context, students educated in an urban environment achieved a significantly higher score in media health literacy (49.22 points; ANOVA, *p* = 0.012), surpassing their peers who were educated in a rural environment (47.29 points).

In addition, students residing in a population of fewer than 500 inhabitants achieved a mean score of 52.16 points in their level of media health literacy, with significant differences (ANOVA, *p* = 0.001) compared to their peers living in larger population centers.

In the same vein, students who balanced their studies with employment demonstrated higher levels of media health literacy (mean score of 50.4 points, with a maximum of 69 points) compared to those who were not employed (mean score of 48.03 points, with a maximum of 66 points), with these differences being statistically significant (ANOVA, *p* = 0.001).

Furthermore, as shown in Table 4, no differences were found in media health literacy levels when this variable was analyzed based on family type (ANOVA, *p* = 0.094), number of siblings (ANOVA, *p* = 0.072), pre-university institution attended (ANOVA, *p* = 0.544), economic level (ANOVA, *p* = 0.222), or self-perceived levels of health and happiness among the students (ANOVA, *p* = 0.176 and *p* = 0.589, respectively).

## 4. Discussion

The objective of this study was to analyze the level of media health literacy among second-year nursing students from five Spanish universities located in diverse regions of the country. With a mean score of 48.73 points, the students demonstrated a deficient level of media health literacy, positioning them, according to the quartile distribution approximation method [30], in the lower part of the distribution (second quartile, below the 50th percentile). Nazarnia et al. [8], who also used the MeHLit scale in a sample of 100 Iranian adults aged 18 to 65 years, obtained a more acceptable result (with a mean score of 55.1 points, they ranked in the third quartile), which was higher than that of the Spanish university students. Similarly, Kim et al. [31] analyzed media health literacy related to smoking among Korean adolescents, reporting average scores of 73.5 points (7.3 points out of 10 on the Primack et al. [32] scale).

The deficient level of media health literacy observed among Spanish university students may be attributed, at least in part, to state university policies that are rife with transversal competencies and lack specific courses for their development [33]. Similarly, the European Higher Education Area (EHEA) has not provided guidance on which specific subjects should incorporate media literacy [14,34]. This situation, combined with shortcomings among faculty (such as inadequate training and low motivation) and a deficit in education funding, has contributed to creating conditions conducive to the development of misinformation in Spain and Europe [35].

The results of the present study suggest the existence of center–periphery inequalities in media health literacy levels in Spain. These differences, which are not absolute, would reveal a gradient of continuity favoring larger population centers. Accordingly, the results showed that students’ scores improved progressively with an increase in the population size of the city where they studied, with the highest scores achieved in Madrid (the largest city in Spain) [36]. This scoring difference compared to other autonomous communities may be explained by several factors, all related to the socio-economic significance of the city’s development. The Autonomous University of Madrid is located in the country’s capital, placing it in an environment of intense economic and cultural activity. This context would facilitate students’ access to more advanced educational resources, allowing its faculties to become leading academic centers for the rest of the nation [37,38]. Furthermore, Madrid serves as a nerve center that hosts both national and international media outlets, making the city a hub for disseminating information, including health-related content [39,40]. Therefore, it seems reasonable that students from the Autonomous University of Madrid present higher levels of media health literacy, as leading institutions—often located in such nerve centers—tend to incorporate innovative approaches in their academic programs [41].

Additionally, it is important to focus on the levels of media health literacy among nursing students at the University of La Rioja (located in the city of Logroño, with a population of 151,294) and those at the University of Extremadura (in the city of Badajoz, with a population of 153,836). The higher scores of the former, with significant differences despite the similar population size [36], indicate the persistence of north–south imbalances within Spain. In this regard, the poorer macroeconomic indicators of the southern regions of Spain [42] and the lower levels of media health literacy reported by students at the University of Extremadura—situated in the southernmost part of the country—are consistent with the report published by Okan et al. [43]. In their work, this group of researchers highlighted the impact of the socio-economic level of the educational environment, which serves as a determinant of the level of media health literacy [43].

In the present study, respondents who reported balancing their studies with paid employment achieved higher levels of media health literacy. Students who work while studying may develop greater resilience and time-management skills, potentially enhancing their competence in searching for and critically evaluating health information. According to Levin-Zamir et al. [44], individuals managing multiple responsibilities, such as combining employment with studies, tend to develop superior information management and informed decision-making skills, which could explain the higher levels of media health literacy observed in this group. Furthermore, it is important to emphasize the internal consistency of all that has been presented thus far, as it is in the larger cities—located in the northern and central regions of Spain—where there is a greater and more diverse job offering. As a result, students in these areas are more likely to be able to balance their studies with paid employment.

In contrast, in the study by Kyaw et al. [45], the authors found that working students had a lower understanding of health information compared to non-working students. The study suggested that added responsibilities and time constraints might hinder adequate literacy. This finding contrasts with others, such as Htay et al. [46], which showed that increased internet use and digital skill development—often correlated with employment—were associated with improved digital health literacy in China. This could also explain the higher levels of media health literacy in employed students in our study. As their digital skills improve, so does their ability to navigate and critically understand the digital media sphere and the health information therein.

It is also important to consider the potential influence of socio-cultural factors, such as Spain’s high unemployment rate, which nearly doubles the European average for those under 25 [47,48]. This context of instability and uncertainty may prompt the early development of additional skills, such as critical thinking, which helps young people identify and anticipate potential risks in the workplace or economy. This critical thinking could also enhance media health literacy, as it equips individuals with the tools necessary to critically analyze health-related media content.

The higher media health literacy scores of students residing in small population centers (fewer than 500 inhabitants) may be interpreted as a contradictory finding. However, this interpretation is far from the reality when viewed through the lens of the “Spain’s depopulated areas” paradigm [49]. From this perspective, the deficit of infrastructure in rural areas necessitates forced mobility for residents to access certain services. These displacements, which would also affect students, often require them to contribute to family expenses through paid activities. Consequently, a profile emerges of a student who, at an early age, faces the abandonment of their family unit, relocation to larger cities in the northern part of the country, and the need to balance their studies with work [50]. Although these conditions may initially seem adverse, they may, in fact, foster the early development of competencies such as greater autonomy and more effective time and academic management [51].

However, students from small population centers may also be more motivated to actively seek accurate health information due to the potential lack of local resources and their reliance on media for health information. Consistent with the study by Yu et al. [52], rural populations rely more heavily on media for information, which has been significantly associated with higher health literacy levels. In this sense, self-efficacy and media can play a key role in eliminating health disparities, encouraging individuals to become more self-taught and effectively use media resources to meet their health information needs, particularly in areas with limited healthcare resources [52,53].

While these findings are significant in our study, they invite future research to examine whether these trends are applicable to other groups of nursing students or if they are specific to the Spanish sample.

Consistent with all that has been discussed, the results of the present study revealed that individuals with higher media health literacy scores were those who also received their pre-university education in an urban environment. This aspect was of interest to González-Cabrera et al. [54], who stated that individuals studying in urban areas had a higher level of media literacy. Academics such as Nutbeam and Lloyd [55] and Pinto Santuber et al. [56] agree that the education received and the environment in which a person grows up are determinants of the competencies and learning strategies that a student will develop throughout their life. In this regard, all data from this study indicate that urban environments and larger population centers in the northern regions are conducive to critical thinking. The development of this competency, driven by the aforementioned socio-economic and socio-educational determinants, could explain the higher levels of media health literacy observed among certain students.

In light of the results obtained in this study, there is an urgent need for public institutions to strategically reorient their action plans, addressing the social inequalities that affect students’ ability to critically analyze health-related information [35]. In this regard, it is important to emphasize that media health literacy among the population serves as a protective factor that reinforces self-care behaviors [57,58].

Similarly, media health literacy also calls upon healthcare professionals to cultivate their symbolic capital in order to be perceived as references in health care management. For this reason, enhancing competencies related to media health literacy in university classrooms is imperative today [59]. Only in this way will students be able to confront a professional future characterized by information overload and the presence of erroneous or blatantly false information that could jeopardize the health of the populations they serve [60].

### Strengths and Limitations of This Study

The study sample consisted of 83.89% women. The gender distribution in our sample consistently reflects the demographic composition of the nursing profession in Spain. According to the Instituto Nacional de Estadística (INE), 84% of nursing professionals in Spain are women [61]. This overrepresentation is consistent with the approximately 80% of women pursuing Health Sciences studies in Spain [62]. The predominance of women is a characteristic of the profession, both nationally and internationally, due to historical and cultural factors that have shaped the perception and choice of nursing as a career [63]. In our study, the proportion of female participants stands at 83.89%, which is consistent with national statistics, further reinforcing the representativeness of our sample regarding gender distribution. However, for future research, it would be beneficial to increase the diversity of the sample. Doing so would not only enable more robust gender studies but also improve the overall validity of the findings. However, as this is an exploratory study, the results provide an initial look at media health literacy among university students who have some health knowledge.

Additionally, this research sheds light on the current skills of students regarding a vital competency like media health literacy, emphasizing the need to integrate it into educational programs. In this way, media health literacy serves as a crucial response to the misinformation problem that is prevalent in our 21st-century digital society, particularly affecting the health sector.

## 5. Implications for Practice

This research provides insight into the influence of socio-economic and socio-educational factors on media health literacy. The poor results observed among nursing students in this competency highlight the need to implement courses focused on developing media health literacy. In this context, healthcare professionals serve as expert references who must have the foundation to critically analyze the health information provided to and shared with the community.

The findings of this study can also guide the development of educational interventions aimed at improving this competency among students. By strengthening critical thinking, future nurses can enhance their ability to communicate effectively with patients, provide guidance, and thereby improve their self-care. Healthcare professionals should take the lead in training the community to critically evaluate the health information they consume, which will influence their lifestyle habits. In this way, community health will benefit from proper health education.

## 6. Conclusions

The sample of nursing students analyzed showed a poor level of media health literacy, with notable differences centered around the north–south and center–periphery axes of the country. Students studying in the larger northern and central cities of Spain achieved higher scores in media health literacy. Consistently, those students who received their pre-university education in an urban environment or who, living in small population centers, were compelled to travel to larger cities, showed higher scores on the MeHLit. This research highlights the positive impact that economic development, employment, and transversal competencies—such as critical analysis—have on media health literacy.

It is urgent for the government and universities to reorient their educational programs to include media health literacy, which should be integrated and assessed as part of the content of the courses included in university degree programs. This measure would enhance the competencies of future health professionals—specifically nurses—who would strengthen their symbolic capital through work with the community and be perceived as genuine references in health and care. In any case, further research is needed to delve deeper into the analysis of media health literacy levels in broader and more diverse groups of health professionals, as well as to conduct multicenter international studies.

## Figures and Tables

**Table 1:** Descriptive analysis of the socio-demographic variables.

Variables	Subtype	N ^1^	Percentage	Mean ± Sd ^2^
Age	-	-	-	21.6 ± 5.9
Gender	Women	349	83.89%	
	Men	65	15.62%	
	Non-binary	2	0.48%	
University of	La Rioja	85	20.43%	
	Zaragoza	118	28.37%	
	Valencia	113	27.16%	
	Extremadura	70	16.83%	
	Autonomous of Madrid	30	7.21%	
Pre-university educational environment	Village	106	25.48%	
	City	310	74.52%	
Number of habitants	<500	6	1.44%	
	500–5000	52	12.26%	
	5000–50,000	129	31.01%	
	50,000–500,000	120	28.85%	
	>500,000	110	26.44%	
Family	Biparental	349	83.89%	
	Homoparental	1	0.24%	
	Monoparental	16	3.85%	
	Divorced parents	41	9.86%	
	Family caregiver	3	0.72%	
	Extended family	6	1.44%	
Number of siblings	One	269	64.66%	
	Two	68	16.35%	
	Three	15	3.61%	
	Four	4	0.96%	
	More than four	7	1.68%	
	None	53	12.74%	
Pre-university education	Public	258	62.02%	
	Private	20	4.81%	
	Subsidized	138	33.17%	
Financial level	Difficulty making ends meet	26	6.25%	
	Enough to make ends meet	124	29.81%	
	More than enough to make ends meet	266	63.94%	
Student Employment	Yes	257	61.78%	
	No	159	38.22%	
Perceived health	Bad	4	0.96%	
	Middling	41	9.86%	
	Good	278	66.83%	
	Very Good	93	22.36%	
Perceived happiness	Very happy	84	20.19%	
	Happy	211	50.72%	
	Moderately happy	107	25.72%	
	Unhappy	14	3.37%	

^1^ N = Population. ^2^ Sd = Standard deviation.

**Table 2:** Mean of media health literacy score (MeHLit-SV) according to gender and university.

Variables		M ^1^	Mdn	SD ^2^	CV ^3^	Min ^4^	Q1 ^5^	Q3 ^6^	Max ^7^	N ^8^	Test
MeHLit-SV score	General	48.73	49	6.90	0.14	29	44	53	69	416	-
Gender											ANOVA *p* = 0.713
	Men	48.63	49	6.89	0.14	30	44	53	69	349	
	Women	49.32	50	7.06	0.14	29	45	55	66	65	
	Non-binary	47.00	47	1.41	0.03	46	46.5	47.5	48	2	
University of	La Rioja	48.20	49	6.69	0.13	32	43	53	65	85	ANOVA *p* = 0.0002
	Zaragoza	49.37	50	7.44	0.15	34	43	55	69	118	
	Valencia	49.97	50	6.60	0.13	30	46	53	68	113	
	Extremadura	45.60	46.5	6.30	0.14	29	42	49	60	70	
	Autonomous of Madrid	50.33	50	5.50	0.10	39	46	54.75	62	30	

^1^ M = mean, ^2^ SD = standard deviation, ^3^ CV = coefficient of variation, ^4^ Min = minimum, ^5^ Q1 = first quartile, ^6^ Q3 = second quartile, ^7^ Max = maximum, ^8^ N = population.

**Table 3:** Mean of media health literacy score (MeHLit-SV) according to pre-university educational environment, number of habitants of the place of residence, and student employment.

Variables		M ^1^	SD ^2^	CV ^3^	Min ^4^	Max ^5^	N ^6^	Test
Pre-university educational environment	Village	47.29	7.39	0.15	29	65	106	ANOVA *p* = 0.012
	City	49.22	6.66	0.13	30	69	310	
Number of habitants	<500	52.16	4.87	0.09	45	60	6	ANOVA *p* = 0.001
	500–5000	46.29	7.03	0.15	32	61	51	
	5000–50,000	47.59	7.30	0.15	29	68	129	
	50,000–500,000	49.75	6.61	0.13	33	65	120	
	>500,000	49.90	6.28	0.12	36	69	110	
Student Employment	Yes	50.40	7.07	0.14	30	69	122	ANOVA *p* = 0.001
	No	48.03	6.71	0.13	29	66	294	

^1^ M = mean, ^2^ SD = standard deviation, ^3^ CV = coefficient of variation, ^4^ Min = minimum, ^5^ Max = maximum, ^6^ N = population.

**Table 4:** Mean of media health literacy score (MeHLit-SV) according to family type, number of siblings, pre-university institution, financial level, and self-perceived health and happiness.

Variables	Subtype	M ^1^	SD ^2^	CV ^3^	Min ^4^	Max ^5^	N ^6^	Test
Family type	Biparental	48.35	6.84	0.14	29	68	349	ANOVA *p* = 0.094
	Homoparental	57.00	6.95	0.12	57	57	1	
	Monoparental	50.50	7.82	0.15	38	69	16	
	Divorced parents	50.46	6.80	0.13	30	61	41	
	Family caregiver	47.00	5.29	0.11	41	51	3	
	Extended family	53.66	5.92	0.11	49	65	6	
Number of siblings	One	48.09	6.80	0.14	29	68	269	ANOVA *p* = 0.072
	Two	49.26	7.57	0.15	32	66	68	
	Three	48.80	7.58	0.15	37	60	25	
	Four	54.25	4.50	0.08	48	58	4	
	More than four	52.14	7.24	0.13	44	65	7	
	None	50.39	5.97	0.11	38	69	53	
Pre-university institution	Public	49.01	7.33	0.14	29	69	258	ANOVA *p* = 0.544
	Private	47.90	6.22	0.12	38	61	20	
	Subsidized	48.31	6.12	0.12	32	65	138	
Financial level	Difficulty making ends meet	49.50	6.30	0.12	36	65	26	ANOVA *p* = 0.222
	Enough to make ends meet	49.51	7.07	0.14	32	69	124	
	More than enough to make ends meet	48.28	6.86	0.14	29	68	266	
Self-perceived health	Bad	49.49	6.78	0.13	35	63	93	ANOVA *p* = 0.176
	Middling	48.22	6.93	0.14	29	68	278	
	Good	50.26	6.85	0.13	38	69	41	
	Very Good	50.50	5.80	0.11	44	58	4	
Self-perceived happiness	Very happy	49.39	7.54	0.15	32	68	84	ANOVA *p* = 0.589
	Happy	48.82	6.51	0.13	32	68	211	
	Moderately happy	48.02	7.26	0.15	29	69	107	
	Unhappy	48.64	5.82	0.11	38	58	14	

^1^ M = mean, ^2^ SD = standard deviation, ^3^ CV = coefficient of variation, ^4^ Min = minimum, ^5^ Max = maximum, ^6^ N = population.

## Data Availability

Detailed data are available upon reasonable request to the corresponding author.

## Public Involvement Statement

No public involvement in any aspect of this research.

## Guidelines and Standards Statement

This manuscript was drafted against the STROBE guidelines for observational studies.
